# Effectiveness of Rituximab, Belimumab, and Thrombopoietin Receptor Agonists in Refractory Immune Thrombocytopenia Associated With Systemic Lupus Erythematosus: A Systematic Review and Meta-Analysis

**DOI:** 10.7759/cureus.99214

**Published:** 2025-12-14

**Authors:** Ryuichi Ohta, Yoshinori Ryu, Kunihiro Ichinose

**Affiliations:** 1 Community Care, Unnan City Hospital, Unnan, JPN; 2 Rheumatology, Shimane University Faculty of Medicine, Izumo, JPN

**Keywords:** belimumab, immune thrombocytopenia, rituximab, systemic lupus erythematosus, thrombopoietin receptor agonists, treatment outcome

## Abstract

Thrombocytopenia is a frequent and clinically significant hematologic manifestation of systemic lupus erythematosus (SLE). A subset of patients develops refractory immune thrombocytopenia (ITP) despite standard therapy, creating a major therapeutic challenge. Targeted agents, including rituximab, belimumab, and thrombopoietin receptor agonists (TPO-RAs), have been used in small studies, but their overall effectiveness in SLE-associated refractory ITP has not been comprehensively evaluated. To systematically review and synthesize evidence on the effectiveness of rituximab, belimumab, and TPO-RAs for refractory ITP in adults with SLE, we conducted a systematic review and meta-analysis of observational studies following a prespecified protocol. Eligible studies included adults with confirmed SLE and refractory ITP treated with rituximab, belimumab, or TPO-RAs. Data were extracted on clinical outcomes, including complete response, partial response, and overall response rate (ORR). A meta-analysis of proportions using fixed-effects models was performed for each treatment category. Methodological quality was assessed using the Newcastle-Ottawa Scale. Eleven studies involving 11 cohorts met the inclusion criteria. Six cohorts (192 patients; pooled ORR 88.5% [170/192]) evaluated rituximab, two cohorts (14 patients; pooled ORR 92.9% [13/14]) evaluated belimumab, and three cohorts (31 patients; pooled ORR 96.8% [30/31]) evaluated TPO-RAs. Rituximab demonstrated a consistently high ORR of 88.5% (170/192) across studies. Belimumab showed an ORR of 92.9% (13/14), although small sample sizes limited evidence. TPO-RAs demonstrated the highest ORR of 96.8% (30/31), with rapid platelet recovery in most patients. Overall study quality was moderate, with larger rituximab cohorts providing the most substantial evidence, whereas belimumab and TPO-RA data were derived from small retrospective studies. Definitions of treatment response varied across studies. Rituximab, belimumab, and TPO-RAs appear to be effective therapeutic options for refractory SLE-associated ITP, with consistently high response rates across available studies. Evidence is strongest for rituximab, while belimumab and TPO-RAs show promising results that require confirmation in larger, well-designed prospective studies. Standardization of response criteria and improved safety reporting are needed to optimize treatment strategies for this challenging clinical condition.

## Introduction and background

Systemic lupus erythematosus (SLE) is a chronic, multisystem autoimmune disease characterized by progressive inflammation affecting multiple organ systems, including the central nervous system, lungs, cardiovascular system, kidneys, and hematologic compartment [1]. Its pathophysiology is largely driven by dysregulation of the adaptive immune system-particularly aberrant B-cell activation, autoantibody production, and immune complex formation [2]. Early diagnosis and appropriate immunosuppressive treatment have improved outcomes; however, disease-related complications and treatment-associated adverse effects continue to contribute substantially to morbidity and mortality [3].

Hematologic abnormalities are among the most common manifestations of SLE, with leukopenia, anemia, and thrombocytopenia frequently observed. Although thrombocytopenia occurs in up to 10-30% of patients with SLE, it is often asymptomatic in the early phases and may be overlooked until platelet counts decline significantly [3]. Severe or persistent thrombocytopenia is clinically important, as it is associated with increased risk of bleeding, hospitalization, and poorer long-term outcomes [4]. Moreover, a subset of patients develop refractory immune thrombocytopenia (ITP) despite standard therapy, including corticosteroids, intravenous immunoglobulin, and conventional immunosuppressants, posing a major therapeutic challenge [4].

In recent years, targeted biologic and thrombopoietin receptor agonist (TPO-RA) therapies have emerged as potential options for refractory SLE-associated ITP [5,6]. Rituximab, a B-cell-depleting anti-CD20 monoclonal antibody, belimumab, an inhibitor of B-cell activating factor (BAFF), and eltrombopag, an oral TPO-RA, have each demonstrated clinical benefit in small observational studies [4,7]. 

Belimumab inhibits BAFF, a cytokine essential for the survival and maturation of autoreactive B cells. BAFF overexpression in SLE contributes to persistent autoantibody production, including antibodies targeting platelets [5,6]. By reducing BAFF signaling, belimumab decreases autoreactive B-cell activity, lowers autoantibody titers, and may thereby mitigate immune-mediated platelet destruction. Additionally, BAFF inhibition has been shown to partially restore megakaryocyte maturation and thrombopoiesis, providing a mechanistic rationale for belimumab’s efficacy in hematologic manifestations, including thrombocytopenia [4,7]. 

However, the effectiveness and safety of these agents in SLE-associated refractory ITP remain insufficiently defined, and no comprehensive systematic review and meta-analysis have synthesized the available evidence to date [6,7]. As SLE populations age and multimorbidity and polypharmacy become increasingly prevalent, the incidence and complexity of thrombocytopenia in routine clinical practice are expected to rise [8]. Establishing evidence-based therapeutic strategies for refractory thrombocytopenia is thus essential for optimizing care [9,10]. Therefore, this study aims to systematically review the literature and quantitatively evaluate the effectiveness of rituximab, belimumab, and eltrombopag in the treatment of refractory immune thrombocytopenia associated with SLE.

## Review

Study design

We conducted this study as a systematic review and meta-analysis of observational studies examining the effectiveness of rituximab, belimumab, and eltrombopag for refractory immune thrombocytopenia associated with SLE. The review was designed and reported in accordance with the Preferred Reporting Items for Systematic Reviews and Meta-Analyses (PRISMA) 2020 guidelines [11]. Before initiating the review process, the study protocol was prospectively registered with the International Prospective Register of Systematic Reviews (PROSPERO; registration ID: CRD420251164996). The primary objective of this review was to synthesize evidence from non-randomized studies to estimate treatment outcomes in this specific, clinically challenging SLE population.

Data sources and search strategy

A comprehensive literature search was performed using three electronic databases: PubMed/MEDLINE, Embase, and Web of Science. The search covered all available publications from database inception to the final search date: September 31, 2025. A combination of controlled vocabulary terms and free-text keywords relating to “systemic lupus erythematosus,” “immune thrombocytopenia,” “rituximab,” “belimumab,” and “thrombopoietin receptor agonists” was employed. Search strategies were tailored to the syntax requirements of each database. Only articles published in English were considered eligible. In addition, reference lists of all included studies were manually screened to identify additional potentially relevant publications.

Eligibility criteria

Studies were selected based on predefined inclusion and exclusion criteria established before screening. Eligible studies were those reporting on adult patients with a confirmed diagnosis of systemic lupus erythematosus and coexisting immune thrombocytopenia that was refractory to at least one standard therapy, such as corticosteroids, intravenous immunoglobulin, or conventional immunosuppressants. To ensure reliability and adequate data extraction, we restricted eligibility to interventional studies and observational studies, including retrospective and prospective cohorts. Studies were required to report treatment with rituximab, belimumab, or eltrombopag and to include clinical outcomes such as complete or partial response, platelet count changes, relapse, or adverse events.

Inclusion Criteria

We included observational studies that reported outcomes for adult patients with refractory SLE-associated immune thrombocytopenia treated with rituximab, belimumab, or thrombopoietin receptor agonists. Studies needed to provide extractable outcome data at either the patient level or aggregated study level and to be published in English. Eligible designs consisted of retrospective or prospective cohorts and multi-patient case series.

Exclusion Criteria

We excluded studies that did not contain original data, including systematic reviews, narrative reviews, letters, editorials, conference abstracts, and commentaries. Single-patient case reports were excluded to minimize reporting bias and enhance the robustness of pooled analyses. Studies were also excluded if thrombocytopenia was not immune-mediated, if the population included only pediatric patients, if SLE diagnosis was uncertain or not clearly defined, or if the intervention did not include one of the three targeted therapies.

Study Selection

All search results were imported into a reference management system, and duplicate records were removed. Two reviewers (RO and YR) independently screened the titles and abstracts of all identified records. Articles deemed potentially relevant were retrieved in full text and evaluated against the eligibility criteria. Disagreements in study selection were resolved through discussion and consensus. The selection process, including the number of records identified, screened, and ultimately included, was documented using a PRISMA 2020 flow diagram.

Data extraction

Data extraction was conducted independently by two reviewers using a standardized and piloted extraction form. Extracted information included publication details, study design, sample size, patient demographics, SLE diagnostic criteria, definitions of refractory thrombocytopenia, prior treatments, and details of the intervention, such as drug dosing and treatment schedule. Clinical outcomes, including complete, partial, and overall response rates, were extracted wherever available. For this review, response outcomes followed definitions used in the original studies: complete response (CR) typically defined as platelet count ≥100×10⁹/L with resolution of bleeding manifestations, partial response (PR) as platelet count 30-100×10⁹/L or at least a two-fold increase from baseline, and overall response rate (ORR) defined as the combined proportion of patients achieving CR or PR. Discrepancies in the extracted data were reconciled by consensus among reviewers. 

Data synthesis

Extracted data were synthesized using both quantitative and qualitative approaches. For studies reporting treatment response, a proportion meta-analysis was conducted to estimate the pooled ORR separately for rituximab, belimumab, and thrombopoietin receptor agonists (TPO-RAs). Because all included studies were single-arm observational designs, and each treatment category comprised a small number of studies, a fixed-effect model was used to calculate pooled estimates with 95% confidence intervals, treating responder counts and total sample size as binomial data. In instances where the reported number of responders exceeded the total sample size due to inconsistent terminology, event counts were capped at the total sample size. Heterogeneity was assessed using the I² statistic and chi-square test; however, meaningful heterogeneity was not anticipated given the methodological similarity across studies.

The methodological quality of included studies was assessed using the Newcastle-Ottawa Scale (NOS) for observational studies [12]. The NOS evaluates three domains, selection, comparability, and outcome, each with a maximum of 3 points. Discrepancies between reviewers were resolved through consensus or consultation with a third reviewer. Studies rated as high quality (NOS ≥ 7) were included in the sensitivity analyses.

Results

Study Selection

**Figure 1:**
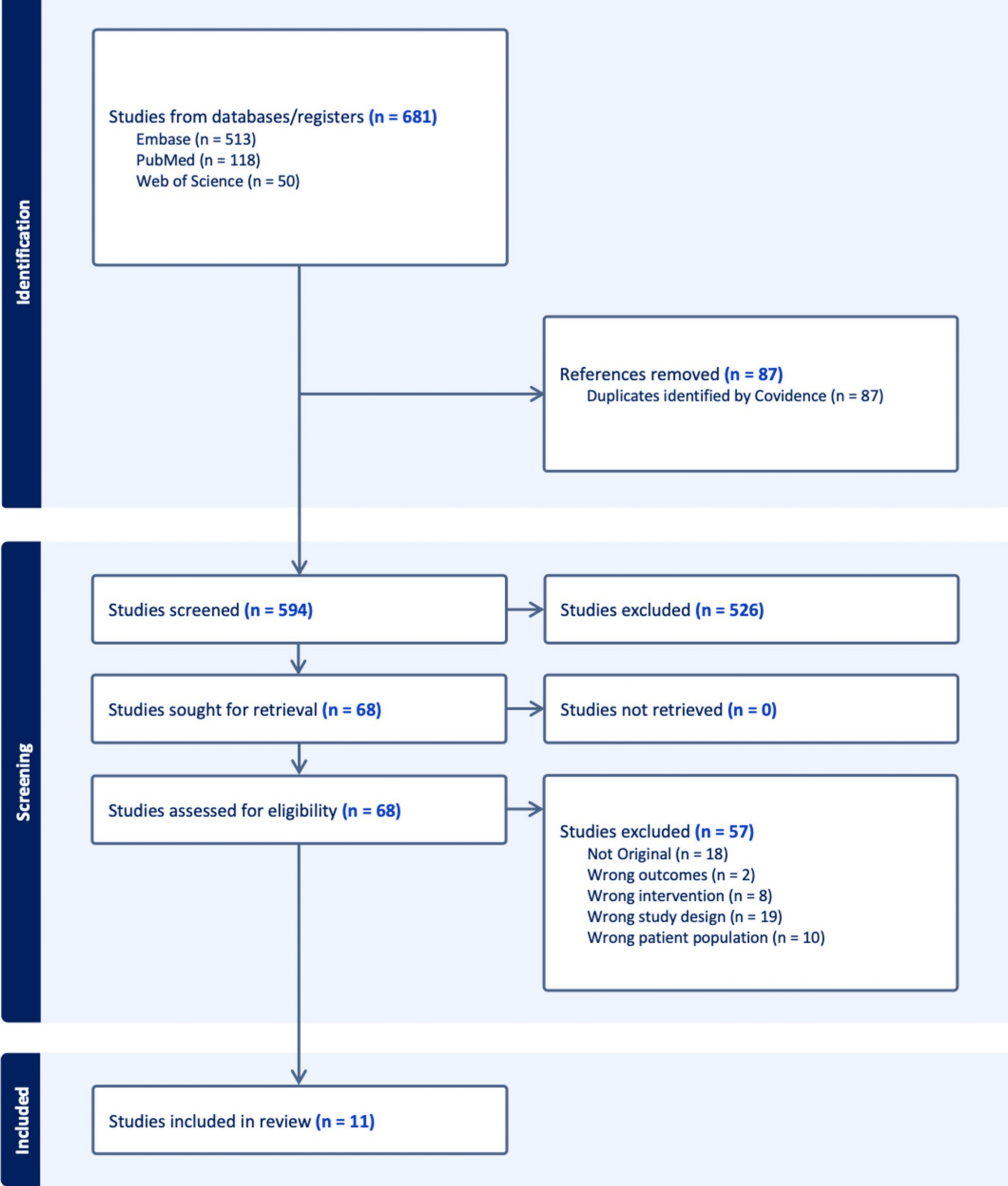
Selection Flow

The initial database search across PubMed/MEDLINE, Embase, and Web of Science identified 681 records, comprising 513 from Embase, 118 from PubMed, and 50 from Web of Science. After removal of 87 duplicate records, 594 unique articles were screened at the title and abstract level. Of these, 526 articles were excluded for failing to meet the eligibility criteria. A total of 68 studies were retrieved for full-text review. Among these, 57 full-text articles were excluded for the following reasons: not original research (n = 18), wrong outcomes (n = 2), wrong intervention (n = 8), inappropriate study design (n = 19), or wrong patient population (n = 10). No studies were excluded due to the inability to retrieve the full text. Ultimately, 11 studies met all inclusion criteria and were incorporated into the final systematic review and quantitative synthesis. A PRISMA 2020 flow diagram summarizing the study selection process is provided in Figure 1.

Characteristics of the Included Articles

A total of 11 observational studies published between 2000 and 2025 were included in this systematic review. All eligible studies were retrospective or prospective cohort studies or multi-patient case series; single-patient case reports were excluded by design. The included studies collectively evaluated the effectiveness of rituximab, belimumab, and eltrombopag in adults with refractory immune thrombocytopenia associated with systemic lupus erythematosus.

Study Designs and Geographical Distribution

Of the 11 included studies, nine studies were retrospective cohort studies, one was a prospective observational study, and one was a multi-patient case series. The studies were conducted across a diverse geographic range, including Asia, North America, and Europe, reflecting variation in clinical practice patterns. Sample sizes ranged widely, from 3 to 71 patients, depending on study design and drug under investigation.

Patient Characteristics

Across the studies, all patients fulfilled established classification criteria for systemic lupus erythematosus (ACR, SLICC, or EULAR/ACR). The mean or median age of participants generally fell within the mid-20s to mid-40s, with a predominance of female patients, consistent with the epidemiology of SLE. Baseline platelet counts were consistently low across all cohorts, confirming that the included populations represented clinically significant thrombocytopenia. All patients had refractory idiopathic thrombocytopenic purpura (ITP), defined variably across studies but typically as inadequate response to corticosteroids, IVIG, and at least one immunosuppressant.

Interventions

Six studies evaluated rituximab, making it the most frequently investigated agent among the included articles. Rituximab dosing regimens varied, with both the lymphoma protocol (375 mg/m² weekly × 4) and the rheumatoid arthritis protocol (1000 mg × 2, two weeks apart) represented. Two studies evaluated belimumab, using either intravenous or subcutaneous formulations according to standard SLE regimens. Two studies investigated eltrombopag, typically administered as an adjunctive therapy following failure of conventional immunosuppressive strategies. One additional study evaluated a combined thrombopoietin-receptor agonist cohort (romiplostim and eltrombopag), but the dataset was synthesized descriptively because drug-specific outcomes could not be isolated (Table 1).

**Table 1:** Demographic data of the included articles Data are presented as reported in each original study. Age is expressed as mean (SD) or median (IQR), as available. NR: not reported. All included studies investigated adult patients with refractory immune thrombocytopenia associated with systemic lupus erythematosus. In the study by Terriou et al., treatment groups consisted of romiplostim alone (six patients; 36%), eltrombopag alone (four patients; 28%), or a combination of both agents (six patients; 36%); individual eltrombopag outcomes could not be isolated and were therefore summarized descriptively [17]. Study designs follow the authors’ original classifications and include retrospective cohort studies, prospective pilot studies, and multi-patient case series.

First author	Year	Country	Design	Sample size	Age (IQR or SD)	Female percentage	Treatment
Chen [13]	2011	China	Prospective pilot study	10	34.3（ 20–42）	100	Rituximab
Jiang [14]	2015	China	Retrospective cohort study	15	37.05 (3.15)	93	Rituximab
Maroun [15]	2015	USA	Retrospective case series	3	46	100	Eltrombopag
Serris [16]	2016	France	Retrospective cohort study	62	36（31–49）	88.7	Rituximab
Terriou [17]	2016	France	Retrospective cohort study	16	NR	75	Romiplostim 36% Eltrombopag 28% Both36%
Serris [18]	2018	France	Retrospective cohort study	71	36（31–48）	85.9	Rituximab
Shobha [19]	2020	India	Retrospective cohort study	12	34.5（30–43）	91.7	Eltrombopag
Zhang [20]	2021	China	Prospective intervention study	8	31.3(7.2)	100	Rituximab
Dong [21]	2024	China	Retrospective cohort study	4	37.8（27–52）	75	Belimumab
Wu [22]	2024	China	Retrospective cohort study	10	34.3(14.0)	100	Belimumab
Dincer [23]	2025	Turkey	retrospective cohort study	26	52.1(14.4)	80.8	Rituximab

Results of Meta-Analysis

A meta-analysis of proportions was performed to estimate pooled ORR for each treatment category: rituximab, belimumab, and thrombopoietin receptor agonists (TPO-RAs; eltrombopag alone or in combination with romiplostim). ORR was defined as the proportion of patients achieving CR or PR. Given the non-comparative nature of the included studies, pooled effects were calculated separately for each intervention using fixed‐effect weighting by sample size, and 95% CIs were derived using the Wilson score method.

**Figure 2:**
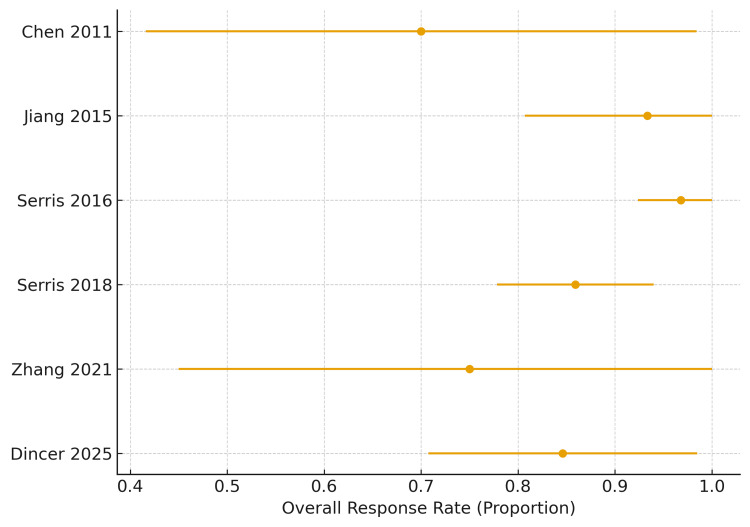
Forest plot of overall response rates for rituximab in refractory SLE-associated immune thrombocytopenia This figure illustrates the overall response rates (ORR) reported across six observational studies evaluating rituximab for refractory immune thrombocytopenia in systemic lupus erythematosus (SLE). Each point represents the study-specific proportion of responders (complete response + partial response), and the horizontal bars denote 95% confidence intervals calculated using binomial methods. Studies are arranged chronologically by year of publication. When reported responder counts exceeded total sample size due to inconsistent terminology, event counts were capped at the total sample size to ensure accurate estimation. Overall, the pooled fixed-effect estimate showed an ORR of 88.5% (170/192; 95% CI 83.3–92.3), demonstrating consistently high response rates across studies despite heterogeneity in study design and rituximab dosing regimens [13,14,16,18,20,23].

Rituximab: Six observational studies, including a total of 192 adult patients with refractory SLE-associated immune thrombocytopenia, evaluated rituximab. Across individual studies, ORR was consistently high, ranging from 70.0% to 100%. In one study where the reported number of responders exceeded the total sample size due to inconsistent response terminology, responder counts were capped at the total sample size to avoid overestimation. The pooled proportion meta-analysis demonstrated a high overall response rate of 88.5% (170/192). The pooled estimate remained robust across both prospective and retrospective designs, with larger French cohorts contributing the most significant weight while showing effect sizes consistent with smaller pilot studies. The 95% confidence interval (83.3%-92.3%)around the pooled ORR indicates a stable and clinically meaningful benefit of rituximab in refractory SLE-associated ITP. Taken together, these findings support rituximab as the most evidence-supported targeted therapy in this setting, although the observational nature of the evidence limits certainty regarding comparative effectiveness (Figure 2).

**Figure 3:**
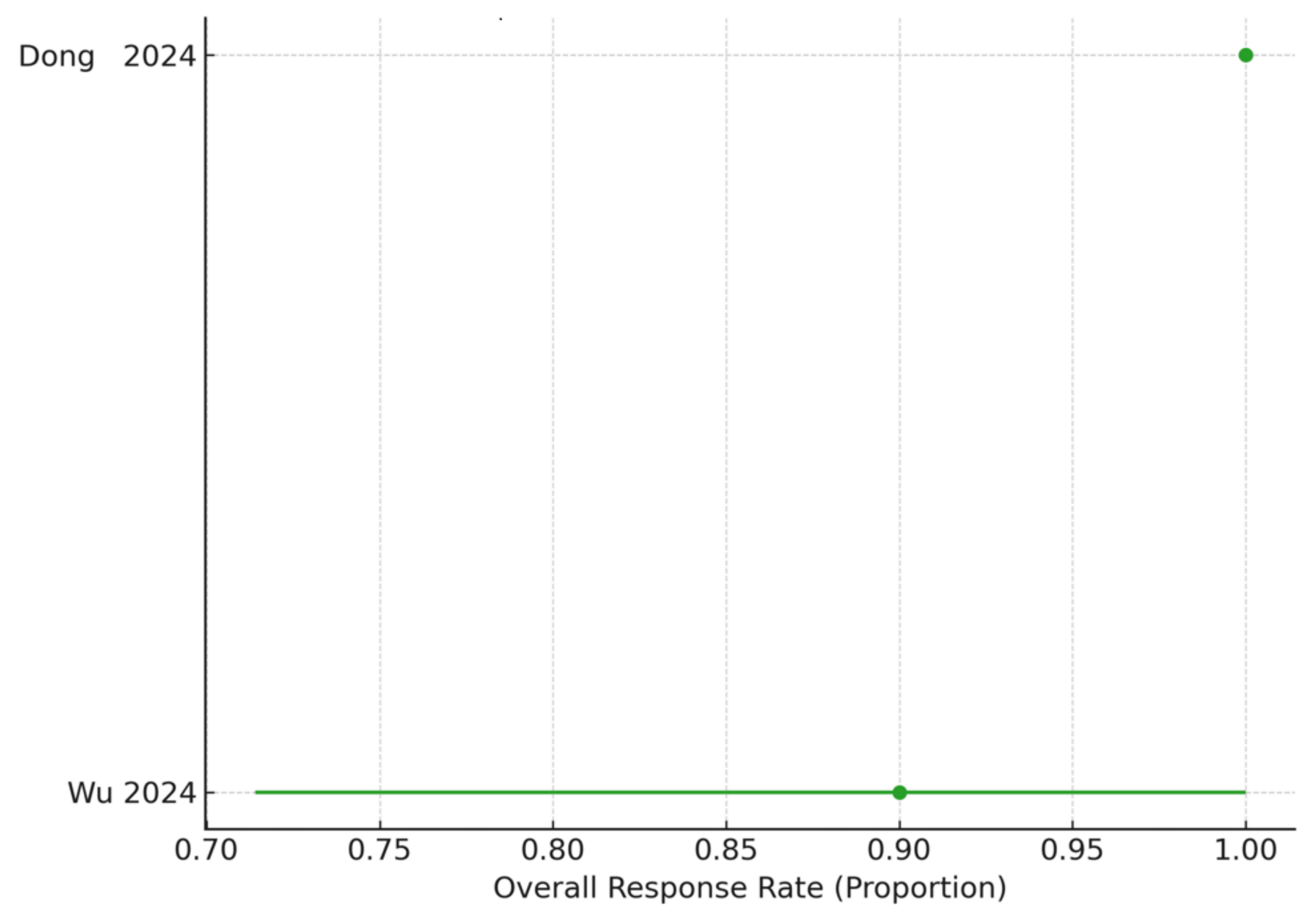
Forest plot of overall response rates for belimumab in refractory SLE-associated immune thrombocytopenia This figure displays the overall response rates (ORR) for belimumab among patients with systemic lupus erythematosus (SLE) and refractory immune thrombocytopenia. Points indicate the study-specific ORR, and horizontal error bars show the corresponding 95% confidence intervals. Both included studies are retrospective cohort analyses and are presented in chronological order. ORR values were extracted from reported complete and partial response outcomes. Although sample sizes were small, both studies demonstrated consistently high treatment responses, with a pooled ORR of 92.9% (13/14) [21,22].

Belimumab: Two retrospective cohorts comprising 14 patients were assessed for belimumab. Both studies reported high response rates, ranging from 90% to 100%. The pooled estimate yielded an ORR of 92.9% (13/14; 95% CI 68.5-98.7). Although the point estimate suggests strong efficacy, the confidence interval was wide, reflecting the very small evidence base and limited precision. Nevertheless, the consistency of high ORRs across both included cohorts supports belimumab as a promising option for refractory SLE-associated thrombocytopenia (Figure 3).

**Figure 4:**
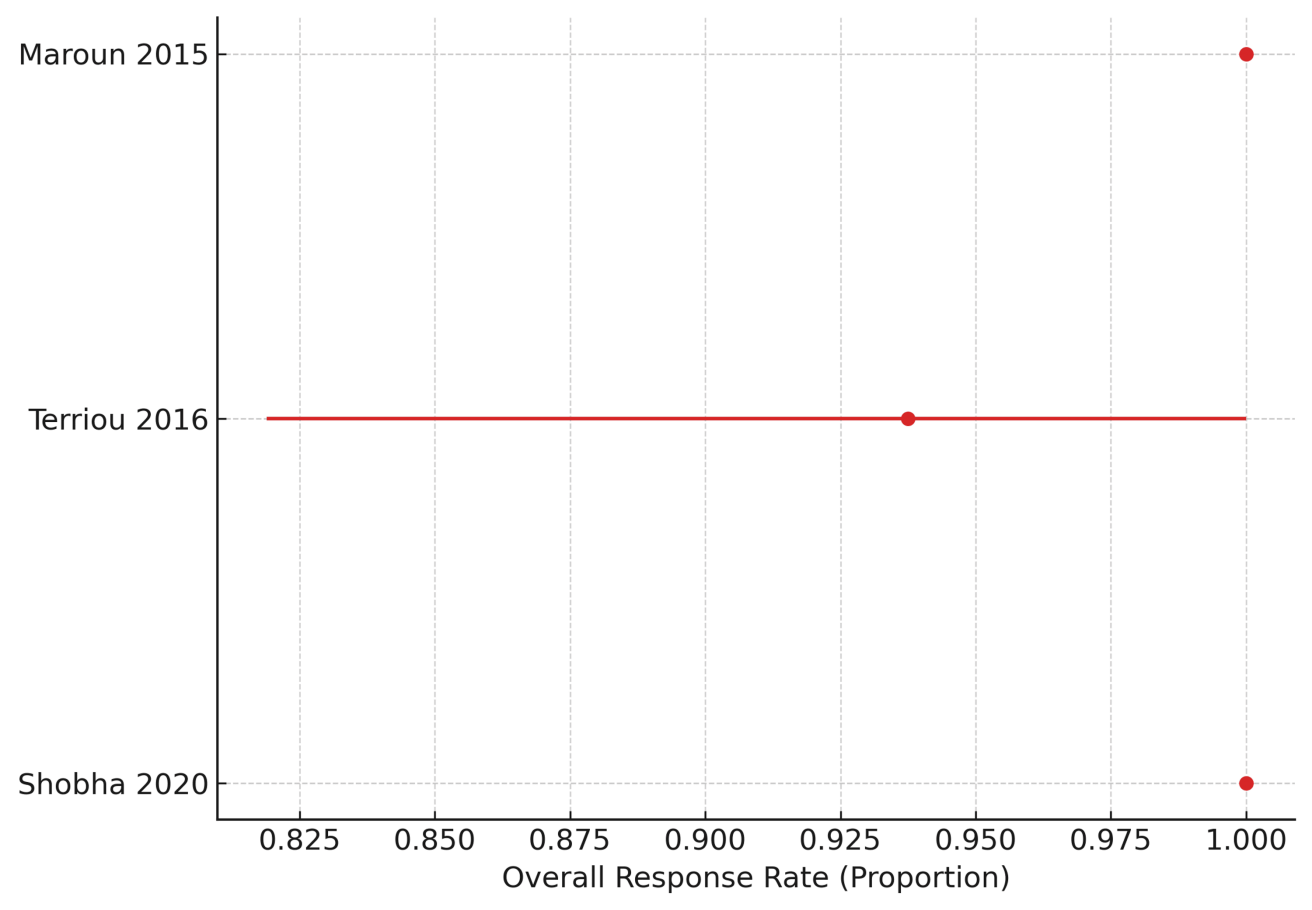
Forest plot of overall response rates for thrombopoietin receptor agonists (eltrombopag ± romiplostim) in refractory SLE-associated immune thrombocytopenia This figure presents overall response rates (ORR) for thrombopoietin receptor agonist (TPO-RA)–based therapy, combining data from eltrombopag monotherapy cohorts and a mixed cohort including romiplostim, eltrombopag, or both. Points represent study-specific ORRs, and horizontal bars denote 95% confidence intervals. Due to the inclusion of a mixed romiplostim/eltrombopag cohort in which treatment effects could not be separated, data are synthesized as a combined TPO-RA category. Studies are ordered by publication year. TPO-RA therapy demonstrated uniformly high ORR across all available studies, with a pooled ORR of 96.8% (30/31), although sample sizes were small [15,17,19].

TPO-RAs (Eltrombopag + Romiplostim mixed): Three studies involving 31 patients were synthesized under the TPO-RA category: two eltrombopag cohorts and one mixed TPO-RA cohort including romiplostim, eltrombopag, or both. Individual ORRs ranged from 93.8% to 100%. The pooled analysis demonstrated an ORR of 96.8% (30/31; 95% CI 83.8-99.4). These findings indicate rapid and highly consistent platelet recovery with TPO-RA-based strategies. However, the pooled estimate should be interpreted cautiously because the evidence is derived from small cohorts, and one study combined two TPO-RAs without isolating eltrombopag-specific effects (Figure 4).

Quality Assessment 

Quality assessment was conducted using the NOS for cohort studies and an adapted version for a small case series. Overall study quality ranged from low to high, reflecting differences in cohort size, representativeness, and outcome reporting. Two large French cohorts by Serris et al. (2016, 2018) achieved the highest methodological quality (9/9), whereas very small studies, such as Dong et al. (2024) and Maroun et al. (2015) were rated low quality due to limited representativeness, absence of comparability, and unclear or non-standardized outcome definitions [15,16,18,21].

In the Selection domain, most cohorts clearly defined SLE-associated immune thrombocytopenia using established classification criteria (ACR 1982/1997 or SLICC 2012) and documented refractory status after standard therapies. Larger cohorts (Serris 2016/2018) were judged representative of routine clinical populations and scored full points. Conversely, small single-center cohorts with limited inclusion detail (Dong 2024; Maroun 2015) were downgraded for potential selection bias and restricted generalizability [15,16,18,21].

In the Comparability domain, the evidence base was constrained by the predominance of single-arm observational designs without control groups. Only studies reporting some adjustment for confounding factors or providing stratified analyses were awarded full comparability stars. Serris 2016/2018 and Dincer 2025 received two stars because they provided broader clinical characterization and more systematic follow-up, whereas studies with no confounder adjustment or explicit comparative framework received one star or none [16,18,23].

In the Outcome domain, most studies used clinically meaningful platelet response criteria (CR/PR/ORR) and reported follow-up sufficient to capture short- to mid-term outcomes. Long follow-up periods in Serris cohorts and Dincer 2025 supported higher outcome scores [23]. Studies lacking clear response definitions (Dong 2024) or with very small sample sizes and heterogeneous reporting (Maroun 2015) were scored lower [15,21].

Taken together, the included evidence is mainly of moderate quality, with strong support from two high-quality cohorts for rituximab. At the same time, belimumab and TPO-RA results rely on smaller observational datasets and should therefore be interpreted cautiously (Table 2).

**Table 2:** Quality Assessment of the Included Studies Using the Newcastle–Ottawa Scale (NOS) This table summarizes the methodological quality of the included observational studies using the Newcastle–Ottawa Scale (NOS) for cohort designs, with an adapted scoring approach applied to the single case series. The NOS evaluates three domains: (1) Selection of study cohorts (maximum 4 stars), (2) Comparability of cohorts based on design or analysis (maximum 2 stars), and (3) Outcome assessment and adequacy of follow-up (maximum 3 stars). Total possible scores range from 0 to 9 for cohort studies and 0 to 6 for case series. Studies with larger and clearly defined cohorts (e.g., Serris 2016 and Serris 2018) achieved the highest scores, reflecting strong representativeness, clear outcome definitions, and adequate follow-up [16,18]. In contrast, smaller studies and case series (e.g., Dong 2024, Maroun 2015) received lower scores due to limited sample size, lack of confounder control, and incomplete reporting [15,21]. Overall, most studies demonstrated moderate methodological quality, consistent with the observational nature of research in refractory SLE-associated immune thrombocytopenia.

First author (Year)	Study design	Selection (0–4)	Comparability (0–2)	Outcome (0–3)	Total NOS score
Chen (2011) [13]	Prospective pilot study	3	1	2	6/9
Jiang (2015) [14]	Retrospective cohort	3	1	2	6/9
Maroun (2015) [15]	Case series (adapted NOS)	2	0	1	3/6
Serris (2016) [16]	Retrospective cohort	4	2	3	9/9
Terriou (2016) [17]	Retrospective cohort	3	1	2	6/9
Serris (2018) [18]	Retrospective cohort	4	2	3	9/9
Shobha (2020) [19]	Retrospective cohort	3	1	2	6/9
Zhang (2021) [20]	Prospective intervention	3	1	2	6/9
Dong (2024) [21]	Retrospective cohort (n=4)	2	0	1	3/9
Wu (2024) [22]	Retrospective cohort	3	1	2	6/9
Dincer (2025) [23]	Retrospective single-centre cohort	3	2	2	7/9

Discussion

Summary of the Study

This systematic review and meta-analysis demonstrated that rituximab, belimumab, and TPO-RAs are effective treatment options for refractory thrombocytopenia in SLE, with pooled overall response rates consistently exceeding 80-90%. These findings suggest that the three agents can overcome poor hematologic outcomes often observed in patients with severe or complicated SLE. However, the interpretation of these results should be approached with caution because the available evidence is limited by small sample sizes, the predominance of young female patients, and the absence of comparative or controlled study designs. Despite these limitations, the present compilation provides a meaningful synthesis of the best available evidence and may guide clinical decision-making in refractory SLE-associated thrombocytopenia.

Comparison with Other Studies

The findings of this review are largely consistent with previous literature. Rituximab and TPO-RAs have well-established benefits in ITP, particularly in cases refractory to corticosteroids and traditional immunosuppressants [24,25]. Their mechanisms of action-B-cell depletion and stimulation of platelet production-align with the known immunopathology of ITP, supporting their application to SLE-associated thrombocytopenia [26].

Conversely, although belimumab has robust evidence demonstrating improvement in global SLE disease activity indices, including SLEDAI and patient-reported outcomes, its specific role in hematologic manifestations has not been thoroughly evaluated [27]. This review could not assess immunologic markers such as anti-platelet antibodies or complement activity because these data were inconsistently reported. Given that the pathophysiology of SLE-associated thrombocytopenia shares key features with primary ITP, it is reasonable to extrapolate treatment strategies across the two conditions [24,25]. Future research should incorporate detailed immunological profiling to clarify treatment response patterns and underlying mechanisms.

Strengths of the Study

This review provides an integrated synthesis of scattered, heterogeneous evidence on novel therapeutics for refractory thrombocytopenia in SLE. By consolidating data from multiple small studies, it provides a clearer overview of treatment effectiveness and helps clinicians navigate therapeutic decisions for a rare and challenging hematologic manifestation of SLE.

Importantly, this study contextualizes treatment responses within the underlying pathophysiology of SLE-associated thrombocytopenia. Unlike ITP, SLE-related thrombocytopenia arises from a combination of immune-mediated platelet destruction-driven by anti-platelet antibodies, immune complex formation, and complement activation-and impaired thrombopoiesis resulting from bone marrow suppression and cytokine dysregulation [28]. These mechanisms provide a biological rationale for the observed benefits of B-cell-directed therapy (rituximab), BLyS inhibition (belimumab), and thrombopoietin receptor agonists, which target autoantibody generation, B-cell survival signals, and megakaryocyte stimulation, respectively [29]. By linking pharmacologic mechanisms with immunopathology, this review underscores why these treatments may be especially effective in refractory disease.

Finally, by identifying patterns across heterogeneous clinical contexts and highlighting gaps in immunologic reporting-such as complement activity, anti-platelet antibodies, and megakaryocyte dynamics-this review not only synthesizes current evidence but also guides priorities for future mechanistic and clinical research [30].

Limitations

Several limitations must be acknowledged when interpreting the findings of this review. First, the evidence base consists entirely of observational studies, primarily retrospective cohorts and small prospective series, without any randomized or head-to-head comparative trials. As a result, causal inference is inherently limited, and treatment effects may be overestimated due to selection bias, regression to the mean, or clinician-driven treatment allocation.

Second, most included cohorts were composed predominantly of young female patients, reflecting the epidemiologic profile of SLE but limiting generalizability to older adults, men, or populations with significant multimorbidity. Older patients with frailty, renal dysfunction, or cardiovascular comorbidities may have different safety profiles or treatment responses, particularly for thrombopoietin receptor agonists, which carry a known thrombotic risk [31]. In the context of SLE, TPO-RAs warrant particular caution because a subset of patients have coexisting antiphospholipid antibodies or established antiphospholipid syndrome, both of which increase thrombotic risk [32,33]. Although the included studies did not report major thrombotic complications attributable to eltrombopag or romiplostim, the small sample sizes may have limited detection of rare events. Previous reports in non-SLE ITP populations have described venous and arterial thrombosis during TPO-RA therapy; therefore, clinicians should assess baseline thrombosis risk and monitor patients closely when using these agents in SLE.

Third, study designs were heterogeneous and, in many cases, of only moderate methodological quality. Clinical settings vary, affecting the results, such as rural and urban settings [4]. Confounding factors, such as baseline SLE disease activity, concomitant immunosuppressant use, serologic profiles (e.g., complement levels, anti-dsDNA), and prior treatment history, were rarely controlled for. This lack of adjustment complicates interpretation of treatment efficacy, as improvements in platelet counts may reflect broader changes in SLE activity rather than drug-specific effects.

Fourth, across the included studies, the definitions of CR, PR, and ORR were not fully standardized. Some studies applied platelet thresholds (e.g., CR ≥100×10⁹/L, PR 30-100×10⁹/L), whereas others used more flexible or unspecified criteria. These discrepancies may introduce variability in response classification and could modestly influence pooled effect estimates. In this review, we relied on each study’s definitions and adjusted event numbers conservatively when inconsistencies were identified; however, the lack of uniform response criteria remains an inherent limitation of the evidence base and may affect comparability between studies. Key secondary endpoints, including the degree and timing of steroid tapering, changes in SLEDAI scores, and normalization of immunologic biomarkers, were heterogeneous or absent, preventing their inclusion in pooled analyses. Additionally, adverse events were reported narratively rather than using standardized toxicity grading, limiting safety assessment.

Finally, small sample sizes, particularly for belimumab (n=14 across two studies) and TPO-RAs (n=30 across three studies), significantly reduce statistical precision and increase the risk of type I and type II errors. Rare but clinically important outcomes, such as thrombosis with TPO-RAs or severe infections with B-cell-directed therapy, may therefore be underdetected. The scarcity of long-term follow-up data further limits conclusions regarding the durability of response and long-term safety.

## Conclusions

Rituximab, belimumab, and thrombopoietin receptor agonists appear to be effective therapeutic options for refractory thrombocytopenia in SLE, with consistently high response rates observed across available studies. Although small and non-comparative study designs limit the current evidence, this systematic review provides a critical synthesis that may support clinical practice. Future prospective, controlled, and mechanistically informed studies are urgently needed to clarify further optimal treatment strategies and patient selection for targeted therapies in SLE-associated thrombocytopenia.
